# Crude Mortality Associated With the Empirical Use of Polymyxins in Septic Patients in a Setting of High Prevalence of Carbapenem-Resistant Gram-negative Bacteria: Retrospective Analysis of a Cohort

**DOI:** 10.1093/cid/ciad272

**Published:** 2023-07-05

**Authors:** Camila L P A M Bezerra, Eusébio L dos Santos, Maura S Oliveira, Maria Beatriz S Dias, Anna S Levin, Maristela P Freire, Icaro Boszczowski, Matias C Salomão

**Affiliations:** Department of Infectious Diseases, Hospital das Clínicas, Universidade de São Paulo, São Paulo, Brazil; Department of Infectious Diseases, Hospital das Clínicas, Universidade de São Paulo, São Paulo, Brazil; Infection Control Department, Hospital das Clínicas, Universidade de São Paulo, São Paulo, Brazil; Infection Control Department, Hospital das Clínicas, Universidade de São Paulo, São Paulo, Brazil; Department of Infectious Diseases, Hospital das Clínicas, Universidade de São Paulo, São Paulo, Brazil; Department of Infectious Diseases, Faculdade de Medicina, Universidade de São Paulo, São Paulo, Brazil; Faculdade de Medicina, Universidade de São Paulo, São Paulo, Brazil; Department of Infectious Diseases, Hospital das Clínicas, Universidade de São Paulo, São Paulo, Brazil; Infection Control Department, Hospital das Clínicas, Universidade de São Paulo, São Paulo, Brazil; Infection Control Department, Hospital das Clínicas, Universidade de São Paulo, São Paulo, Brazil; Department of Infectious Diseases, Faculdade de Medicina, Universidade de São Paulo, São Paulo, Brazil

**Keywords:** sepsis, antibiotic therapy, antimicrobial resistance, polymyxin

## Abstract

**Background:**

Our aim in this retrospective cohort study was to assess the impact on mortality of the empirical use of polymyxin as therapy for carbapenem-resistant gram-negative bacteria (CR-GNB) in septic patients. The study was performed at a tertiary academic hospital in Brazil, from January 2018 to January 2020, the pre–coronavirus disease 2019 period.

**Methods:**

We included 203 patients with suspected sepsis. The first doses of antibiotics were prescribed from a “sepsis antibiotic kit”, which contained a selection of drugs, including polymyxin, with no preapproval policy. We developed a logistic regression model to assess risk factors associated with 14-day crude mortality. Propensity score for polymyxin was used to control biases.

**Results:**

Seventy (34%) of 203 patients had infections with at least 1 multidrug-resistant organism isolated from any clinical culture. Polymyxins in monotherapy or in combination therapy were prescribed to 140 of the 203 (69%) patients. The overall 14-day mortality rate was 30%. The 14-day crude mortality was associated with age (adjusted odds ratio [aOR], 1.03; 95% confidence interval [CI], 1.01–1.05; *P* = .01), SOFA (sepsis-related organ failure assessment) score value (aOR, 1.2; 95% CI, 1.09–1.32; *P* < .001), CR-GNB infection (aOR, 3.94; 95% CI, 1.53–10.14; *P* = .005), and time between suspected sepsis and antibiotic administration (aOR, 0.73; 95% CI, .65–.83; *P* < .001). The empirical use of polymyxins was not associated with decreased crude mortality (aOR, 0.71; 95% CI, .29–1.71; *P* = .44).

**Conclusions:**

Empirical use of polymyxin for septic patients in a setting with high CR-GNB prevalence was not associated with decreased crude mortality.

## Teaser

In this retrospective cohort study, we found that the empirical use of polymyxin in septic patients was not associated with a decrease in crude mortality, even in a setting with a high prevalence of carbapenem-resistant gram-negative bacteria.

Globally, approximately 49 million cases of sepsis and septic shock occur each year [1]. These are medical emergencies, and early identification and proper management are essential to reduce morbidity and mortality [2, 3]. Several studies have shown improved survival with early antimicrobial therapy and increased mortality proportional to the delay in treatment, especially in septic shock [4–6]. However, clinicians must balance the early administration of antimicrobials with the harm that results from the inadequate use of antibiotics, including unnecessary use in patients without infection [7].

Antimicrobial resistance may increase due to the use of broad-spectrum antibiotics as empirical therapy in patients with sepsis or septic shock [7, 8]. Resistance leads to difficult-to-treat infections, longer hospital stays, increased morbidity and mortality, and has an impact on the global economy [8, 9].

Some antibiotic-resistant organisms, such as carbapenem-resistant *Acinetobacter baumannii* (CRAB), carbapenem-resistant *Pseudomonas aeruginosa* (CRPA), and carbapenem-resistant Enterobacterales (CRE), are of major concern due to the severity of infection and the spread of resistance, leading to few treatment options [10–12].

Treatment of CRE infections, particularly in Brazil, is often restricted to the use of drugs such as polymyxins, aminoglycosides, tigecycline, or fosfomycin. In low- to middle-income and low-income countries, the “new antibiotics,” such as ceftazidime-avibactam, are available in only 8% of them [13]. In addition to the high cost of these drugs, limited access to laboratory techniques for identifying carbapenemases makes their use difficult [14]. Since their reintroduction into clinical practice, polymyxins have been used as salvage therapy for multidrug-resistant gram-negative bacterial infections caused by *P. aeruginosa*, *A. baumannii*, and Enterobacterales [15–19].

At our hospital, due to the high prevalence of extended-spectrum beta-lactamase–producing gram-negative bacteria, meropenem is suggested as empirical treatment of hospital-acquired sepsis, and polymyxins are recommended when there is an increased risk of infection with organisms resistant to carbapenems. A 2017 study at our hospital linked exposure to healthcare in the previous year, liver disease, and antibiotic use to the increased risk for CRE colonization [20].

Our aim in this study was to evaluate the impact of the initial use of polymyxin as therapy for carbapenem-resistant gram-negative bacteria (CR-GNB) on mortality in patients with suspected or confirmed sepsis.

## METHODS

### Study Design and Sample Analysis

We conducted a retrospective cohort study in hospitalized patients with suspected or confirmed sepsis from 2 January 2018 to 11 January 2020. This study was conducted at the Central Institute of Hospital das Clinicas, an 800-bed tertiary teaching hospital affiliated with the University of São Paulo, Brazil. We analyzed 14-day crude mortality to assess the impact of the empirical use of polymyxin on septic patient survival.

### Study Participants

The cohort consisted of all patients aged ≥16 years with suspected sepsis who received the first dose of antibiotic from the “sepsis antibiotic kit” during the study period. The sepsis antibiotic kit is a ready-for-use antibiotic kit for administration of the first dose of antibiotics; it does not require preapproval by the Hospital Infection Control Team if the patient meets criteria for sepsis outlined in the 2012 sepsis guidelines [21]. The sepsis antibiotic kit contains ceftriaxone, metronidazole, oxacillin, meropenem, vancomycin, and polymyxin B. The choice of initial empirical antibiotic is made by the clinician based on the suspected infection and local epidemiology (Supplementary Material). The patients were evaluated from the moment of suspected sepsis until hospital discharge or death. Patients who died within 48 hours of suspected sepsis were excluded.

### Data Collection and Processing

For the analysis of appropriateness of antibiotic therapy, we considered only patients whose infection had an isolated infectious agent. An antimicrobial treatment regimen was defined as appropriate if the bacteria isolated were susceptible in vitro to the antibiotic prescribed (Clinical and Laboratory Standards Institute, 2021) and the spectrum was the narrowest possible [22]. Regimens were classified as monotherapy or combination therapy depending on the number of active drugs included.

An infection that occurred at least 72 hours after hospital admission or that was related to an invasive procedure performed during the current hospitalization was defined as healthcare-associated. We defined previous hospitalization, previous surgery, previous immunosuppression, and previous use of antibiotics as those that occurred 90 days prior to the current hospital admission.

The primary outcome of interest was all-cause mortality within 14 days of the diagnosis of sepsis. The main exposure variable was initiation of empirical use of polymyxin at the time of sepsis suspicion. Patients who were already on antibiotics at the time of sepsis suspicion were not excluded from the study.

The following variables were abstracted from electronic medical records: sex; age; comorbidities (using the Charlson comorbidity index [23]); previous hospitalization; previous surgery; previous immunosuppression; previous or current use of antibiotics; length of hospital stay before suspected sepsis and unit in which the infection was treated; clinical data (temperature, cardiac frequency, respiratory frequency, blood pressure, mental status, oxygen therapy); laboratory data (leukocyte and platelet counts, creatinine, serum lactate); SOFA (sepsis-related organ failure assessment) score [24] on the day of sepsis diagnosis; infection site; multidrug-resistant organism (MDRO) infection; CR-GNB infection; initial empirical use of meropenem, polymyxin, and vancomycin; and time between suspected sepsis and initial antibiotic administration.

We also evaluated MDRO colonization or infection after resolution of the initial sepsis through weekly colonization screening for CRE, CRPA, and CRAB of patients admitted to the intensive care unit (ICU; according to institutional protocol) and of clinical samples collected later for a reason other than the initial diagnosis of sepsis.

The MDROs considered in this study were CRE, CRPA, CRAB, methicillin-resistant *Staphylococcus aureus* (MRSA), and vancomycin-resistant *Enterococcus* spp. (VRE). The CR-GNB considered in this study were CRE, CRPA, and CRAB.

### Statistical Analyses

We compared survivor and nonsurvivor groups to identify predictors of 14-day crude mortality.

We used *χ*^2^ or Fisher exact tests to compare dichotomous variables, and we used the Mann–Whitney test to compare continuous variables. Variables with a *P* value < .2 in the bivariate analysis were included in the multivariate analysis, which was performed by backward stepwise regression and was used to identify independent risk factors. We initially included highly correlated variables, but they were excluded in the backward selection, and only the one that best fit the model was kept for analysis. A 2-tailed *P* value < .05 was considered significant.

Because polymyxin may have been prescribed in a population of patients with specific characteristics, which would introduce a bias into the analysis, we use a propensity score to balance the baseline characteristics of patients in the exposed (ie, those receiving polymyxin) and unexposed groups and to adjust risk factors for 14-day crude mortality.

We estimated the propensity score of receiving polymyxins. The variables included in this calculation were previous hospitalization, previous surgery, hospital stay days before suspected sepsis, opening the protocol in the ICU, more than 10% of young neutrophil forms, and initial empirical use of meropenem. The area under the receiver operating characteristic curve for the final model was 0.72 (95% confidence interval [CI], .65–.80; Supplementary Material). The propensity score was used in 2 ways: as a covariable in logistic regression analysis and to construct an inverse probability of treatment weighting cohort. For the multivariate analysis of risk factors for 14-day crude mortality and for the calculation of the propensity score for the use of polymyxin, we considered only CR-GNB infections.

We estimated a sample size of 205 patients to detect a decrease in 14-day mortality from 50% to 30% when polymyxin was used in the antimicrobial treatment, considering a 2:1 inclusion ratio (137 with polymyxin and 68 without polymyxin), with an 80% power and alpha of 0.05. Analysis was performed using STATA version 16.1 (StataCorp, College Station, TX).

The Hospital das Clínicas’ Ethics Committee approved this study.

## RESULTS

Of the 203 patients included in this study, 153 (75%) were men with a mean age of 52 years (range, 17–93). Fifty-one patients (25%) had a previous hospitalization, and 130 patients (64%) had a previous surgical procedure. Most patients (187, 92%) received antibiotics during the previous 90 days, and most were using antibiotics at the time of the suspected sepsis (119, 59%). Two hundred patients (99%) had healthcare-associated infections. Among the types of infection, bloodstream infection was the most frequent with 60 cases (30%), followed by pneumonia (26%, 52 cases) and abdominal infection (12%, 25 cases). Of the 3 community-onset infections, 2 were skin and soft tissue infections and 1 was osteomyelitis. The main characteristics of the patients are included in Table 1.

**Table 1.** Baseline Characteristics, Clinical Data, and Outcomes of 203 Septic Patients: Brazil, 2018–2020

Baseline Characteristic	Overall N = 203 (%)	Received Polymyxin n = 140 (%)	Did Not Receive Polymyxin n = 63 (%)	*P* Value
Age, mean (IQR), y	52.3 (17–93)	53 (17–92)	55 (21–93)	.6
Male sex, n (%)	153 (75)	105 (75)	49 (78)	.6
Charlson comorbidity index, mean (IQR)	3 (0–11)	3 (0–11)	2 (0–10)	.5
Previous hospitalization,^a^ n (%)	51 (25)	42 (30)	11 (17)	.05
Previous surgery,^a^ n (%)	130 (64)	96 (68)	34 (54)	.05
Previous immunosuppression,^a^ n (%)	51 (25)	42 (30)	10 (16)	.04
Previous use of antibiotics,^a^ n (%)	187 (92)	131 (93)	54 (86)	.08
Current antibiotic use, n (%)	119 (59)	…	…	
Previous hospital stay, mean (IQR), d	5 (0–240)	…	…	
Suspected sepsis treatment in the intensive care unit, n (%)	189 (93)	134 (96)	57 (90)	.03
Clinical characteristics on the day of suspected sepsis				
Temperature ≥38°C or ≤36°C, n (%)	168 (83)	…	…	
Heart rate ≥90 bpm, n (%)	180 (89)	…	…	
Systolic blood pressure ≤90 mm Hg or mean arterial pressure ≤65 mm Hg, n (%)	154 (76)	…	…	
Respiratory rate ≥20 bpm, n (%)	132 (65)	…	…	
Oxygen therapy, n (%)	168 (83)	…	…	
Altered mental status (Glasgow coma scale <14), n (%)	102 (50)	…	…	
Leukocytes ≥12 000/mm³ or ≤4000/mm³, n (%)	148 (73)	…	…	
More than 10% of young neutrophil forms (band cell and/or neutrophilic metamyelocyte), n (%)	44 (22)	…	…	
Platelets (×10³/mm³), mean (IQR)	299 (1–4.510)	…	…	
Creatinine >1.2 mg/dL, n (%)	137 (68)	…	…	
Serum lactate >14 mg/dL, n (%)	121 (60)	…	…	
SOFA score, mean (IQR)	9 (1–21)	9 (1–21)	9 (1–18)	.9
Source of infection				
Community-acquired, n (%)	3 (1)	…	…	
Healthcare-associated, n (%)	200 (99)	…	…	
Infection site^b^				
Bloodstream infection, n (%)	60 (30)	…	…	
Pneumonia, n (%)	52 (26)	…	…	
Abdominal infection, n (%)	25 (12)	…	…	
Urinary tract infection, n (%)	17 (8)	…	…	
Central nervous system infection (acute bacterial meningitis, brain abscess, ventriculitis), n (%)	14 (7)	…	…	
Skin and soft tissue infection, n (%)	14 (7)	…	…	
Febrile neutropenia, n (%)	7 (3)	…	…	
Osteomyelitis, n (%)	2 (1)	…	…	
Surgical site infection, n (%)	1 (0.50)	…	…	
Tracheobronchitis, n (%)	1 (0.50)	…	…	
Unknown source, n (%)	10 (5)	…	…	
Patients with MDRO infection, n (%)	70 (34)	56 (40)	7 (11)	.9
MDRO isolated in clinical cultures, n	78	…	…	
Carbapenem-resistant gram-negative bacteria isolated in clinical cultures, n	58	…	…	
Carbapenem-resistant *Klebsiella pneumoniae*, n	25	…	…	
Carbapenem-resistant *Acinetobacter baumannii*, n	21	…	…	
Carbapenem-resistant *Pseudomonas aeruginosa*, n	10	…	…	
Other carbapenem-resistant Enterobacterales, n	2	…	…	
No isolated microorganism, n (%)	77 (38)	77 (38)	…	-
Clinical outcomes				
14-day crude mortality, n (%)	60 (30)	41 (29)	42 (67)	.5
In-hospital crude mortality, n (%)	113 (56)	…	…	
Colonization or infection by MDROs after the resolution of the initial sepsis, n (%)	109 (54)	85 (61)	25 (40)	.009

Abbreviations: IQR, interquartile range; MDRO, multidrug-resistant organism; SOFA, sepsis-related organ failure assessment.

Previous hospitalization, previous surgery, previous immunosuppression, and previous use of antibiotics are those that occurred 90 days prior to the current hospital admission.

Infection sites are not mutually exclusive.

The median time between the initial diagnosis of sepsis and antibiotic administration was 40 minutes (range, 0–1131). The median time from sepsis suspicion to initiation of antibiotics for a susceptible pathogen was 13 hours (range, 0–168), including empirical or definitive therapy.

Overall 14-day mortality was 30%, and overall in-hospital mortality was 56%. Most patients were severely ill with a mean SOFA score of 9 (range, 1–21).

At the time of suspected sepsis, 140 (69%) patients received polymyxins alone or in combination with other antibiotics. The median time of polymyxin use in the survivor group was 8 days (range, 1–72); in the nonsurvivor group, it was 7 days (range, 1–23).

The following variables were included in the multivariate analysis: age, Charlson comorbidity index, previous hospitalization, previous use of antibiotic, current use of antibiotic, previous hospital stay days, axillary temperature, altered mental status, more than 10% of immature neutrophil forms (band cells and/or neutrophilic metamyelocyte), oxygen therapy, serum creatinine and lactate, platelets, SOFA score, infection caused by a CR-GNB, empirical use of polymyxin, time between suspected sepsis and antibiotic administration, and propensity score. The factors significantly associated with 14-day crude mortality in the multivariate analysis after adjusting for propensity score were increased age, increased SOFA score, infection caused by a CR-GNB, and time between suspected sepsis and antibiotic administration (Table 2).

**Table 2.** Bivariate and Multivariate Analysis for 14-Day Mortality in Patients With Suspected Sepsis

Variable	Survivors (n = 143) n (%)	Nonsurvivors (n = 60) n (%)	Bivariate Analysis		Multivariate Analysis Propensity Score as Covariable		Multivariate Analysis Using Inverse Probability Weighted Cohort	
			RR (95% CI)	*P* Value	aOR (95% CI)	*P* Value	aOR (95% CI)	*P* Value
Male Female	109 (76) 34 (24)	44 (73) 16 (27)	1.04 (.8–1.3)	.7	…			
Age, mean (IQR), y	50 (17–89)	59 (23–93)	…	.002	1.03 (1.01–1.05)	.01	1.02 (1.00–1.06)	.03
Charlson comorbidity index, mean (IQR)	3 (0–11)	4 (0–10)	…	.01	…		…	
Previous hospitalization,^a^ n (%)	30 (21)	21 (35)	1.8 (.9–3.4)	.08	…		…	
Previous surgery,^a^ n (%)	91 (64)	39 (65)	1.01 (.8–1.2)	.9	…		…	
Previous immunosuppression,^a^ n (%)	35 (24)	16 (27)	1.03 (.8–1.3)	.7	…		…	
Previous use of antibiotic,^a^ n (%)	134 (94)	53 (88)	0.8 (.5–1.2)	.2	…		…	
Current use of antibiotics, n (%)	77 (54)	42 (70)	1.2 (1.02–1.4)	.03	…		…	
Previous hospital stay, mean (IQR), d	4	6	…	.1	…		…	
Suspected sepsis treatment in the intensive care unit, n (%)	131 (93)	58 (97)	2.2 (.5–10.4)	.3	…		…	
Axillary temperature ≥ 38°C, n (%)	97 (68)	32 (53)	0.8 (.7–1.01)	.05	…		…	
Axillary temperature ≤36°C, n (%)	21 (15)	18 (30)	1.4 (1.02–1.9)	.01	…		…	
Oxygen therapy, n (%)	113 (79)	55 (92)	1.3 (1.07–1.5)	.04	…		…	
Altered mental status, n (%)	70 (50)	32 (53)	1.3 (.9–1.7)	.08	…		…	
More than 10% of young neutrophil forms (band cell and/or neutrophilic metamyelocyte), n (%)	28 (19)	16 (27)	1.1 (.9–1.5)	.3	…		…	
Platelets (×10³/mm³), mean (IQR)	326.4 (1–4510)	235.1 (9–1200)		.05				
Creatinine value >1.2 mg/dL, n (%)	89 (62)	48 (80)	1.3 (1.06–1.5)	.01				
Serum lactate value >14 mg/dL, n (%)	78 (55)	43 (72)	1.2 (1.04–1.5)	.02				
SOFA score, median (IQR)	8 (1–21)	11 (3–20)	1.1 (1.06–1.2)	<.001	1.2 (1.09–1.32)	<.001	1.15 (1.07–1.32)	.006
Initial empirical use of meropenem, n (%)	75 (52)	32 (53)	1.01 (.9–1.2)	.9				
Initial empirical use of vancomycin, n (%)	86 (60)	38 (63)	1.04 (.9–1.3)	.7				
Initial empirical use of polymyxin, n (%)	101 (71)	39 (65)	0.9 (.8–1.1)	.4	0.71 (.29–1.71)	.44	0.74 (.84–1.11)	.61
Time between suspected sepsis and antibiotic administration, mean (IQR), min	107 (0–1131)	67 (0–418)	…	.2	0.73 (.65–0.83)	<.001	0.76 (.62–0.84)	.002
Patients with carbapenem-resistant gram-negative bacteria infection, n (%)	41 (29)	29 (48)	1.3 (1.05–1.6)	.007	3.94 (1.53–10.14)	.005	3.52 (1.56–11.83)	.005
Propensity score	…	…	…		2.07 (.18–23.79)	.56		

Abbreviations: aOR, adjusted odds ratio; CI, confidence interval; IQR, interquartile range; SOFA, sepsis-related organ failure assessment.

Previous hospitalization, previous surgery, previous immunosuppression, and previous use of antibiotics are those that occurred 90 days prior to the current hospital admission.

**Figure 1.**
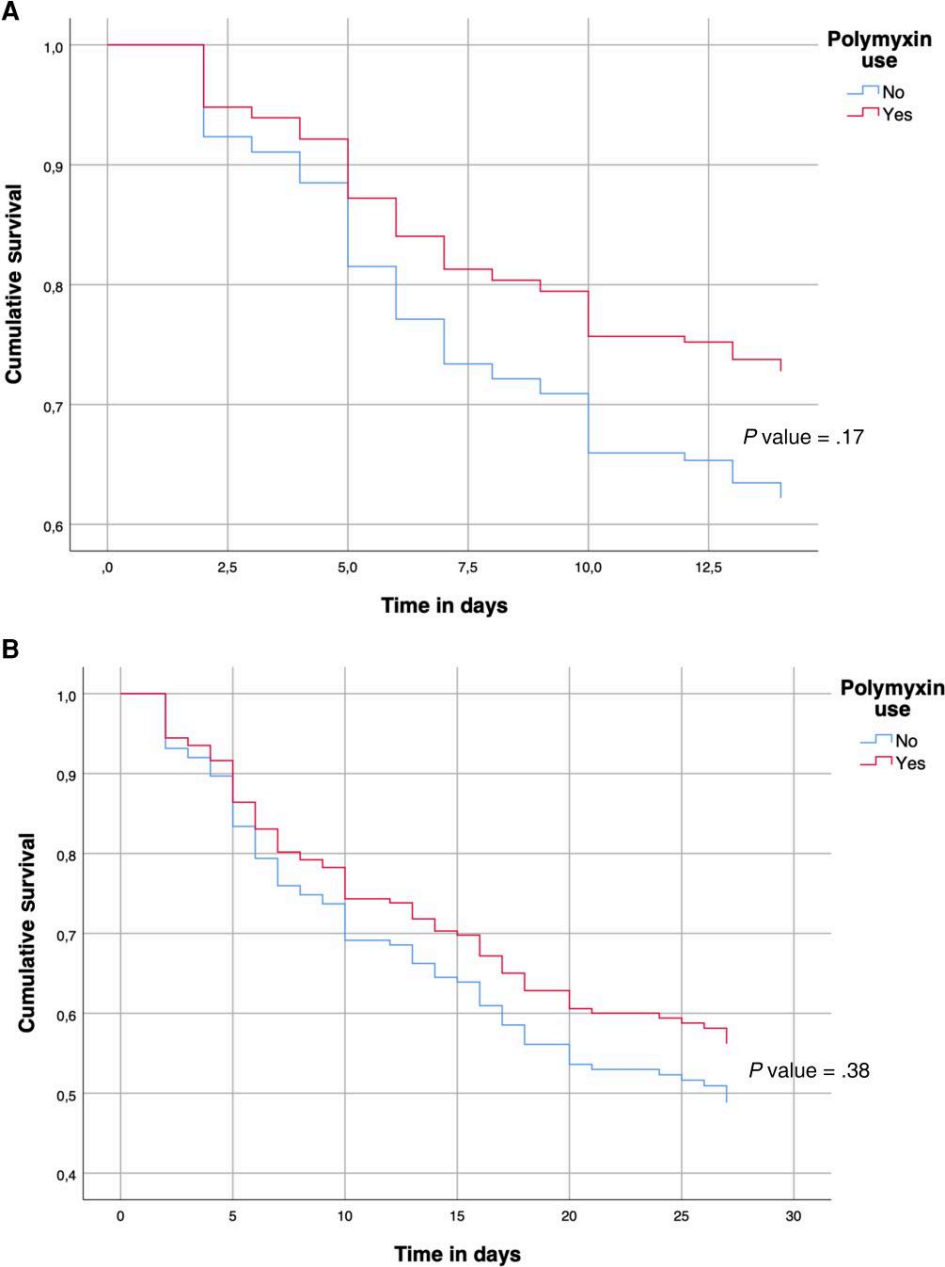
Empiric use of polymyxin.

The empirical use of polymyxin was not associated with crude mortality reduction in the multivariate or in the 14- and 30-day survival analyses (Table 2, Figure 1).

Table 1 compares baseline characteristics between patients who received polymyxin and patients who did not receive polymyxin.

All patients had at least 1 clinical sample collected for culture, but 77 patients (38%) had no isolated infectious agent in these samples. Of these patients, 51 (66%) discontinued antibiotic therapy. Ninety-three (74%) of the 126 patients who had a pathogen isolated had a pathogen that was susceptible to the empirical therapy prescribed. Twenty-nine percent of those empirical treatments had an adequate antibiotic spectrum, and 71% had a spectrum broader than necessary. For definitive therapy, 114 (90%) of the 126 patients had a pathogen isolated that was susceptible to the prescribed antibiotics, but 17% of these patients received antibiotics with a broader than necessary spectrum.

Antibiotics were escalated after pathogen identification in 18 of 126 patients (14%), and antibiotics were deescalated in 68 of 126 patients (54%), with a total of 96 of 126 patients being switched from multiple antibiotic therapy to monotherapy (76%).

Seventy of 203 patients (34%) had infections with at least 1 MDRO isolated from clinical specimens. Of all microorganisms isolated from clinical cultures, 78 were MDRO. Carbapenem-resistant *Klebsiella pneumoniae* was the most frequent, with 25 of 78 isolates (32%), 7 of which were polymyxin-resistant (28%). Twenty-one of 78 isolates (27%) were CRAB, 10 of 78 isolates (13%) were CRPA, and 2 of 78 (2.5%) isolates were other CRE. Among gram-positive bacteria, there were 11 of 78 isolates (14%) of MRSA and 9 of 78 isolates of VRE (11.5%).

A total of 109 patients (54%) developed MDRO colonization or infection after the initial diagnosis of sepsis.

## DISCUSSION

In this retrospective cohort of patients with suspected or confirmed sepsis in a tertiary teaching hospital, we evaluated the impact of the initial use of polymyxin as empirical therapy for CR-GNB on mortality. We observed that the use of polymyxin empirically was not associated with a decrease in 14-day crude mortality despite the high prevalence of CR-GNB in the hospital (median of CRE colonization pressure in the ICUs of 21%) [25].

A few studies on the use of polymyxins for the treatment of severe infections from multidrug-resistant bacteria in Brazil have suggested the suboptimal efficacy of these antibiotics [26–28]. Our study is important because it expands the scientific evidence on the empirical use of polymyxins in clinical practice, especially in septic patients in low-income countries. Most of the studies target polymyxin for treating known multidrug-resistant bacteria, but there is a lack of knowledge on the effects of empirical polymyxin use [26–28].

The empirical use of polymyxins was also not associated with 14-day or 30-day survival. However, the group that used empirical polymyxins demonstrated a nonstatistical trend for better survival using the sample size calculated for this study. This finding raises the question of what sample size would be necessary to find a significant difference and what would be the number of patients needed to treat with polymyxin in order to reduce mortality. Polymyxins are toxic drugs and are associated with many adverse events; renal toxicity is one of the most important. A recent meta-analysis showed that the prevalence of nephrotoxicity during polymyxin treatment in the ICU was 34.8%, similar to the non-ICU setting [29]. We should consider the balance between the potential benefit of liberally using empirical polymyxins and the risk of severe adverse events.

The 14-day crude mortality rate found in our study was lower than expected. However, the in-hospital mortality was still high (56%), similar to the findings in another study conducted in Brazilian ICUs, with a sepsis-associated mortality rate of 56% [30]. A systematic review of new definitions of septic shock from Sepsis-3 showed an in-hospital mortality rate associated with septic shock of 46.5% [3].

The association between the use of early antibiotic therapy and reduced mortality appears to be stronger in patients with septic shock than in patients with sepsis without shock [4, 6, 31, 32]. Thus, in line with the Surviving Sepsis Campaign 2021, the need and timing for initiation of antibiotic therapy in patients with suspected sepsis should be guided by the likelihood of infection and the severity of illness [7]. In our study, the time between suspected sepsis and antibiotic administration was associated with a decrease in mortality.

We found that 95% of the patients initially used broad-spectrum antibiotics at the time of the clinical diagnosis of sepsis, that is, they used vancomycin and/or polymyxin alone or combined with other antimicrobials. The choice for broad-spectrum antibiotics in the initial empirical therapy could be justified by the presence of risk factors for MDRO infections, such as hospital-acquired infections or previous healthcare exposure, antibiotic use in the past 90 days, and local prevalence of MDROs [33–37]. In our study, most patients had healthcare-associated infections (99%) and had used antibiotics previously (92%). The majority of patients (200, 99%) were already hospitalized when they developed sepsis; however, we still had 3 (1%) patients who were septic in the emergency department.

We found a prevalence of MDRO infection of 34% in our hospital, with a predominance of carbapenem-resistant *K. pneumoniae*. Twenty-eight percent of isolates in clinical cultures of *K. pneumoniae* were resistant to polymyxin. Although the use of broad-spectrum antibiotics may be necessary for empirical therapy in septic patients, adjustments after microbiologic results are important to spare unnecessary use and reduce toxicity. Among the patients who received broad-spectrum antibiotic therapy, 54% developed MDRO colonization or infection after the resolution of their sepsis.

In order to minimize inappropriate empirical use of polymyxins, we suggest restricting their use only for patients at high risk for CR-GNB infections, according to local hospital epidemiology and other factors such as previous hospitalization history, previous CRE colonization, and use prior use of carbapenems, or for targeted culture-guided therapy [20]. We believe that polymyxin should be available as a ready-for-use antibiotic in the sepsis kit of our hospital, especially for definitive antibiotic therapy, considering our high prevalence of resistance to carbapenems and limited economic resources.

The new antibiotics (eg, ceftazidime-avibactam, ceftolozane-tazobactam) are the first-line therapy for severe CRE and CRPA infections per the most recent guidelines. However, these drugs are not broadly available in Brazil and other low- and middle-income countries, and it is important to ensure access to them, including to the diagnostics and stewardship needed for their proper use [17–19]. In addition to limited access to these new antibiotics in Brazil, mainly due to the high cost, it is important to consider that in a scenario in which CRAB is the second most frequent pathogen, the empirical use is even more uncertain. Thus, the incorporation of newer and faster diagnostic technologies for identification of pathogens and carbapenemases can be a tool for the correct use of available antibiotics. The implementation/improvement of antimicrobial stewardship interventions for review of broad-spectrum antibiotic therapy is also important.

Our study has limitations. This was a retrospective single-center study, which limits generalization of results. We had to consider the time of prescription of antimicrobials as the time of the clinical diagnosis of sepsis and the time of checking the medications in the electronic medical record as the time of antibiotic administration, which may have led to an underestimation of the number of hours between diagnosis and start of antibiotic therapy. We could not rule out infection for patients who did not have an infectious agent isolated. Consequently, we did not consider those patients for analysis of empirical and definitive antibiotic therapy. The patient selection process was not randomized, and the negative association between polymyxin and survival might have been influenced by an unmeasured variable. The study sample was small, unbalanced, and observational, so unmeasured confounders may persist despite the use of multivariate analysis. We did not assess polymyxin-related adverse events in these patients. We did not assess prior MDRO colonization and infection and did not describe the types of multidrug-resistant organisms associated with infection or colonization after sepsis treatment.

In conclusion, we did not find an association between empirical use of polymyxin and improved survival for patients with suspected or confirmed sepsis in a setting of high MDRO prevalence. Continued efforts, including appropriate use of antibiotics, access to newer antibiotics, and improved diagnostic methods, are needed to reduce mortality in our population.

## Supplementary Data

Supplementary materials are available at *Clinical Infectious Diseases* online. Consisting of data provided by the authors to benefit the reader, the posted materials are not copyedited and are the sole responsibility of the authors, so questions or comments should be addressed to the corresponding author.

## Supplementary Material

ciad272_Supplementary_Data

## Notes

***Acknowledgments.*** The authors are grateful to the Infection Control Team that provided the sepsis protocol sheets for data collection in this study. They thank Ana Rubia Guedes Vinhole, research nurse, and Luizegne Donato, from the Grupo de Controle de Infecção Hospitalar do Hospital das Clínicas, for helping with the data collection. They also thank Fernanda Lessa, acting chief of the International Control Program, Division of Healthcare Quality Promotion, Centers for Disease Control and Prevention (CDC), and Benjamin Park, senior technical advisor with the Global Fund, CDC, for revising the manuscript.

***Supplement sponsorship.*** This article appears as part of the supplement “The Evolving Challenges of Antibiotic Resistance in Low- and Middle-Income Countries: Priorities and Solutions,” sponsored by the U.S. Centers for Disease Control and Prevention, and Health Security Partners.
