# Supplementary material for: Crude Mortality Associated With the Empirical Use of Polymyxins in Septic Patients in a Setting of High Prevalence of Carbapenem-Resistant Gram-negative Bacteria: Retrospective Analysis of a Cohort
Source: Clin Infect Dis. 2023 Jul 5;77(Suppl 1):S62–9. doi: 10.1093/cid/ciad272 (PMC10321694; doi:10.1093/cid/ciad272)
Supplement: ciad272_Supplementary_Data [file ciad272_supplementary_data.docx]

**Supplementary material**

**Title: Crude mortality associated with the empirical use of polymyxins in septic patients in a setting of high prevalence of carbapenem-resistant Gram-negative bacteria: retrospective analysis of a cohort**

Authors: Camila L. P. A. M. Bezerra¹, Eusébio L. dos Santos Jr.¹, Maura S. Oliveira², Maria Beatriz S. Dias², Anna S. Levin^1,3,4^, Maristela P. Freire¹, Icaro Boszczowski², Matias C. Salomão^2,3^

1. Department of Infectious Diseases, Hospital das Clínicas, Universidade de São Paulo, São Paulo, Brazil;
2. Infection Control Department, Hospital das Clínicas, Universidade de São Paulo, Brazil;
3. Department of Infectious Diseases, Faculdade de Medicina, Universidade de São Paulo, Brazil;
4. Faculdade de Medicina, Universidade de São Paulo, Brazil;

**Corresponding author:**

Matias C. Salomão, M.D., Ph.D.

Av Dr Enéas De Carvalho Aguiar, 255, 4º Andar, Department of Infectious Diseases - Cerqueira César, São Paulo, SP, Brazil.

E-mail address: matias.salomao@hc.fm.usp.br

**Alternate Corresponding author:**

Camila L. P. A. M. Bezerra, M.D.

Av Dr Enéas De Carvalho Aguiar, 255, 4º Andar, Department of Infectious Diseases - Cerqueira César, São Paulo, SP, Brazil.

E-mail address: camila.bezerra@hc.fm.usp.br

**Contents:**

1. **Supplementary Table 1 - p. 4**
2. **Supplementary Table 2 - p. 5**

**Supplementary Table 1.  Factors associated with the use of polymyxin for calculation of the propensity score (PS) to adjust risk factors for 14-day mortality.**

| **Variables** | **Coefficient (95% IC)** | **p-Value** |
| --- | --- | --- |
| **Previous hospitalization** | 0.6 (0.1 - 1.1) | 0.01 |
| **Previous surgery** | 0.4 (-0.03 - 0.8) | 0.07 |
| **Hospital stay days before suspected sepsis** | 0.05 (0.01 - 0.08) | 0.009 |
| **Opening the protocol in the intensive care unit** | -0.5 (-1 - 0.08) | 0.09 |
| **More than 10% of young neutrophil forms** | -0.5 (-0.9 - -0.001) | 0.05 |
| **Initial empirical use of meropenem** | -0.6 (-0.9 - -0.2) | 0.003 |

**Supplementary Table 2.  Recommendations of the Instituto Central of the Hospital das Clínicas of the University of São Paulo for the empirical treatment of sepsis.**

| **Suspected infectious focus** | **Community-acquired infection** | **Healthcare-associated infection*** | **Patient colonized by MDRO**^a^ **and/or use of carbapenems in the last 90 days** |
| --- | --- | --- | --- |
| **Pulmonary** | Ceftriaxone + Azithromycin | Meropenem + Vancomycin** | Meropenem + Polymyxin B + Vancomycin** |
| **Urinary** | Ceftriaxone | Amikacin | Amikacin |
| **Abdominal** | Ceftriaxone + Metronidazole | Meropenem +/- Vancomycin** | Meropenem + Polymyxin B +/- Vancomycin** |
| **Gastroenterocolitis** | Ciprofloxacin + Metronidazole | Ciprofloxacin + Metronidazole +/- Vancomycin orally*** | Ciprofloxacin + Metronidazole +/- Vancomycin orally*** |
| **Spontaneous bacterial peritonitis** | Ceftriaxone | Piperacillin-tazobactam | Meropenem + /- Polymyxin B |
| **Skin and soft parts** | Ceftriaxone + Oxacillin | Meropenem + Vancomycin | Meropenem + Polymyxin B + Vancomycin |
| **Fournier syndrome** | Clindamycin + Amikacin + Ceftazidime | Meropenem + Vancomycin | Meropenem + Polymyxin B + Vancomycin |
| **Gas gangrene or post trauma injury or accident with venomous animals** | Ceftriaxone + Clindamycin | - | - |
| **Bloodstream infection associated with central venous catheter** | - | Meropenem + Vancomycin | Meropenem + Polymyxin B + Vancomycin |
| **Meningitis or ventriculitis** | Ceftriaxone + Ampicillin**** | Meropenem + Vancomycin | Meropenem + Polymyxin B + Vancomycin |
| **Post-chemotherapy febrile neutropenia** | - | Piperacillin-tazobactam + Vancomycin | Meropenem + Polymyxin B + Vancomycin |
| **Chorioamnionitis or puerperal infection (endometritis)** | Ampicillin-sulbactam or Clindamycin + Gentamicin | Ampicillin-sulbactam or Clindamycin + Gentamicin | Ampicillin-sulbactam or Clindamycin + Gentamicin |
| **Undefined focus** | Ceftriaxone | Piperacillin-tazobactam + Vancomycin | Meropenem + Polymyxin B + Vancomycin |

**^a^MDRO: multidrug-resistant organisms.**

***Hospitalization or surgery in the last 90 days, patients with a long-term catheter attending chemotherapy/dialysis clinics/day hospital with catheter manipulation in the last 30 days.**

****Consider coverage for methicillin-resistant Staphylococcus aureus if the patient underwent surgery in the last 90 days or had a long-term catheter that had been manipulated in the last 30 days.**

*****Previous use of antibiotics or previous confirmed Clostridioides difficile infection.**

******Patients over 60 years of age or immunosuppressed.**
